# Correlation between crown-rump length in the first trimester of pregnancy and neonatal outcomes

**DOI:** 10.1186/s12887-022-03426-8

**Published:** 2022-07-01

**Authors:** Yin Xu, Meng Ni, Qianqian Zhang, Jiuru Zhao, Zheng Tang, Zhiwei Liu

**Affiliations:** 1 International Peace Maternity and Child Health Hospital, School of Medicine, Shanghai Jiao Tong University, Shanghai, China; 2 International Peace Maternity and Child Health Hospital, China Welfare Institution, Shanghai, China; 3 Shanghai Key Laboratory of Embryo Original Disease, Shanghai, China; 4 Department of Neonatology, International Peace Maternity and Child Hospital, School of Medicine, Shanghai Jiao Tong University, 910# Hengshan Road, Shanghai, 20030 China

**Keywords:** CRL, Neonatal outcomes, SGA, LGA, Preterm

## Abstract

**Background:**

To investigate the association of crown-rump length (CRL) during the first trimester of pregnancy with neonatal outcomes.

**Methods:**

A total of 15,524 women with a reliable first day of the last menstrual period and a regular menstrual cycle (28 ± 4 days) were included from January 2015 to November 2016. CRL was measured by ultrasound from 7^+0^ to 13^+6^ weeks during pregnancy and transformed to a standard deviation score (SDS) adjusted for gestational age. Linear regression was used to explore risk factors for CRL. A generalised linear model was used to evaluate the association between CRL and neonatal outcomes.

**Results:**

In the multivariate analysis, maternal age (0.25 mm, 95% CI = [0.22–0.28], *P* < 0.001; 0.04 SDS, 95% CI = [0.03–0.04], *P* < 0.001), multipara (0.30 mm, 95% CI = [0.08–0.52], *P* = 0.007; 0.04 SDS, 95% CI = [0.00–0.07], *P* = 0.031) and folic acid supplement use (0.78 mm, 95% CI = [0.49–1.08], *P* < 0.001; 0.05 SDS, 95% CI = [0.01–0.10], *P* < 0.019) were positively associated with CRL, while pre-pregnancy BMI (-0.17 mm, 95% CI = [-0.21 to -0.13], *P* < 0.001; -0.02 SDS, 95% CI = [-0.03 to -0.02], *P* < 0.001) was negatively related to CRL. For neonatal outcomes, CRL was negatively associated with small for gestational age (SGA) ([risk ratio] (RR) = 0.733, 95% [CI] = 0.673–0.8004, *P*_adjusted_ < 0.001) and neonatal intensive care unit (NICU) admission ([RR] = 0.928, 95% [CI] = 0.883–0.976, *P*_adjusted_ = 0.003), and preterm birth ([RR] = 1.082, 95% [CI] = 1.008–1.162, *P*_adjusted_ = 0.029), but positively related to large for gestational age (LGA) ([RR] = 1.241, 95% [CI] = 1.184–1.301, *P*_adjusted_ = 0.012). When stratified by pre-pregnancy BMI, the risk of SGA and LGA remained significant in all groups, while the increased risk of preterm birth was only observed in the lean group (BMI < 18.5 kg/m^2^) and decreased risk of NICU admission rate in the normal group (BMI 18.5–24 kg/m^2^).

**Conclusions:**

Maternal characteristics were independently associated with CRL in the first trimester, which was negatively related to foetal size, SGA, preterm birth, and admission rate to the NICU, but positively related to LGA.

**Supplementary Information:**

The online version contains supplementary material available at 10.1186/s12887-022-03426-8.

## Background

Crown-rump length (CRL), measured using ultrasound as early as the first prenatal visit, is generally used to assess gestational age in the first trimester [1]. It is also performed as a part of aneuploidy screening, accompanied by measurements of nuchal translucency thickness and biochemical markers [2–4]. A growing number of studies have shown that prenatal complications might originate from conditions in the very early stages of pregnancy, such as embryo implantation [5, 6], when a malfunctional placenta, genetic heterogeneity, and nutrition contribute to different growth patterns in the first trimester [7, 8]. Thus, CRL might be an early and useful indicator of foetal growth and other neonatal conditions.

Most studies investigated the role of CRL in the assessment of foetal size such as birth weight, small for gestational age (SGA) and large for gestational age (LGA), which has a close relationship with foetal, neonatal, and adult health [9, 10]. For example, researchers found that SGA is a risk factor for cerebral palsy, psychological disorders, and poor intellectual performance in term and moderate to late preterm infants [11–14]. For LGA, infants are exposed to long-term metabolic complications credibly, including childhood obesity [15], and the metabolic syndrome in their adulthood [16]. However, owing to the limited sample size, other neonatal outcomes have not yet been elucidated.

In this study, we investigated the correlation between CRL in the first trimester and neonatal outcomes in natural singleton pregnancies to offer insights for the early recognition of adverse outcomes [17].

## Methods

### Study participants

**Fig. 1:**
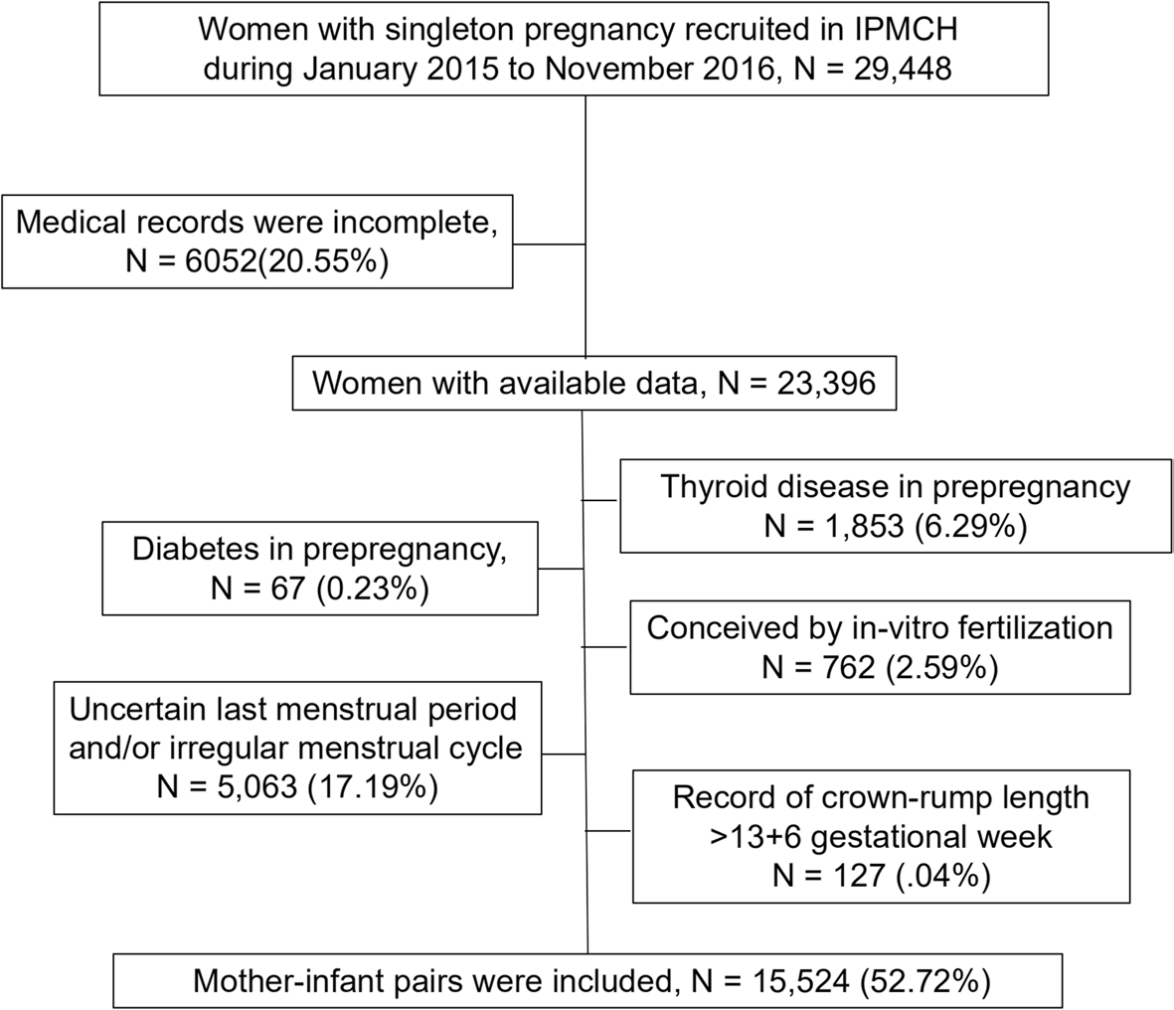
Flowchart of the participants included in the analysis

Women who underwent antenatal assessment and delivered at the International Peace Maternity and Child Health Hospital (IPMCH) in Shanghai between January 2015 and November 2016 were enrolled in the study. A total of 29,448 women with natural singleton pregnancies were included. After excluding 6,052 cases without complete medical records, 67 with pre-pregnancy diabetes, 1,853 with a history of thyroid disease, and 762 with in vitro fertilization, 20,714 mother-infant pairs remained. The gestational age was calculated based on the last menstrual period. Subsequently, 5,063 women with neither a known first day of the last menstrual period nor a regular menstrual cycle of 28 ± 4 days were excluded. To investigate CRL in the first trimester, 127 cases of CRL measured beyond 14 weeks of gestation were excluded. Ultimately, 15,524 mother-infant pairs with confirmed gestational ages of 7–13^+6^ weeks were included (Fig. 1).

### Foetal ultrasonography

All foetal biometry measurements were recorded by qualified technicians according to the clinical guidelines recommended by the International Society of Ultrasound in Obstetrics and Gynecology (ISUOG) [9]. Standard ultrasound machines (Philips, Netherlands) equipped with real-time, grayscale, two-dimensional (2D) transducers, a freeze frame, and electronic calipers were used in the study.

### Terms and definitions

All circumstances were confirmed in accordance with the clinical protocols and the International Classification of Diseases, 10^th^ Revision, Clinical Modification.

1. Maternal characteristics: gestational diabetes (all women undergo mandatory gestational diabetes screening between 24–28 weeks of gestation; any of the following criteria are met at a 75 g oral glucose tolerance test: fasting: ≥ 92 mg/dL, 1 h: ≥ 180 mg/dL, 2 h: ≥ 153 mg/dL); intrahepatic cholestasis (pruritus, elevated serum total bile acid [> 10 μmol/L] and/or alanine aminotransferase), and pre-eclampsia (high blood pressure and excess protein in the urine after 20 weeks of pregnancy). The serum vitamin D concentration was described in our previous study [18].
2. Neonatal outcomes: preterm delivery (a live birth before 37 weeks of gestation); SGA (birth weight < 10th percentile of newborns at the same gestational age), LGA (birth weight > 90th percentile of newborns at the same gestational age), using birth weight distribution of live births stratified by gestational age in Shanghai as a standard; admission to NICU; asphyxia (a failure to initiate or sustain spontaneous breathing at birth), wet lung (chest X-ray symptoms: alveolar, interstitial, interlobar pleural or pleural cavity effusion, emphysema), hyperbilirubinemia (serum bilirubin concentrations higher than 220 μmol per litre in full term infants, 255 μmol per litre in preterm infants), necrotising enterocolitis (systemic signs and intestinal signs, with or without radiological features), sepsis (haemodynamic changes and other clinical manifestations with isolation of a pathogen), and still birth (for any reason). All neonatal outcomes were recorded during hospitalisation.

### Statistical analysis

All statistical analyses were performed using the SPSS (version 25, IBM Corp., Armonk, NY, USA) and R (version 4.1.0) software packages. The Kolmogorov–Smirnov test was used to test the normality of the variables. One-way ANOVA or the Kruskal–Wallis test was applied to compare multiple groups, with Bonferroni corrections for post hoc tests. For missing data on age and BMI, multiple imputations were applied for analyses, and no significant effect was found in the sensitivity analysis. CRL were transformed to SDS adjusted for gestational age using “GAMLSS” package by R (Figure S1). The characteristics of the SDS prediction models are listed (Table S1).

Second, we used linear regression models adjusted for foetal sex and gestational age to separately assess the relationship between covariates and CRL. The results are represented as the effect size of the actual CRL and SDS for each outcome. Subsequently, all the factors associated with CRL were included in the multivariate linear regression model.

Third, we examined the associations between CRL and neonatal outcomes (birth length, birth weight, SGA, LGA, preterm birth, and admission to the NICU) using a generalised linear model adjusted for maternal age, pre-pregnancy BMI, gestational age, foetal sex, mode of delivery, parity to generate risk ratios (RRs), and 95% confidence intervals (CIs). When multiple comparisons were performed, the significance level was adjusted using the Bonferroni correction. Statistical significance was set at *P* < 0.05 (two tailed).

### Ethics approval and consent to participate

This study was performed in accordance with the relevant local guidelines and regulations. The study protocol was approved by the Medical Ethical Committee of the IPMCH (No. GKLW2012-49), School of Medicine, Shanghai Jiao Tong University, and this study was registered to the Chinese Clinical Trial Registry (registration number: ChiCTR1900027447). Written informed consent was obtained from all the participants.

## Results

### Characteristics of mother-infant pairs

In total, 15,524 mother-infant pairs were included in the study. The maternal and neonatal characteristics are described in Table 1. Of the entire population, the average maternal age was 30.4 ± 3.7 years, and the pre-pregnancy BMI was 21.0 ± 2.6 kg/m^2^. Of the women, 63.7% were primiparous. A total of 14.2% of the participants consumed supplementary folic acid. The average vitamin D concentration in serum was 42.97 ± 15.85 mmol/L. During pregnancy, 10.3% of the women were diagnosed with gestational diabetes mellitus, 2.4% with preeclampsia, and 0.7% with intrahepatic cholestasis of pregnancy. A total of 51.7% of newborns were male, and the average gestational age was 38.9 ± 1.4 weeks.

**Table 1:** Clinical Characteristics of the Study Population (*n* = 15 524)

**Clinical Characteristics of the mother-infant pairs**		**CRL Standard Deviation Score (Mean** ± **SD)**	***P* value**
**Gestational age when CRL was recorded (weeks) (Mean ± SD)**	11.82 ± 0.74		
**Maternal Age (years) (Mean ± SD) (%)**^**abc**^	30.4 ± 3.7		< .001
< 25 (3.7)		-0.17 ± 1.02	
25–35 (87.3)		-0.04 ± 0.99	
> 35 (9.0)		0.28 ± 0.99	
**Pre-pregnancy BMI (kg/m**^**2**^**) (Mean ± SD) (%) **^**bc**^	21.0 ± 2.6		< .001
< 18.5 (13.3)		0.02 ± 0.98	
18.5–24 (75.6)		0.02 ± 0.99	
≥ 24 (11.1)		-0.15 ± 1.06	
**Primipara (%)**	9896(63.7)		< .001
1		-0.04 ± 0.99	
≥ 2		0.06 ± 1.01	
**Folic acid supplement use**	2212(14.2)		
No		-0.01 ± 1.01	
Yes		0.04 ± 0.95	
**Vitamin D concentration (nmol/L) (Mean ± SD)**^**a**^	42.97 ± 15.85		0.001
< 50(67.6)		-0.02 ± 1.01	
50–75(29.4)		0.02 ± 0.99	
> 75(8.1)		0.14 ± 0.95	
**Family history of diabetes (%)**	1253 (0.08)	-	
**Family history of thyroid disease (%)**	8	-	
**Gestational diabetes mellitus (%)**	1594 (10.3)	-	
**Preeclampsia (%)**	378 (2.4)	-	
**Intrahepatic cholestasis of pregnancy (%)**	109 (0.7)	-	
**Delivery mode**			0.446
Spontaneous delivery	8934 (57.6)	-0.01 ± 1.00	
cesarean	6588 (42.4)	0.01 ± 0.99	
**Male fetus (%)**	8025 (51.7)		< .001
Male		0.05 ± 1.00	
Female		-0.05 ± 1.00	
**Apgar score (Mean ± SD)**	9.89 ± 0.65	-	
**Gestational age at delivery (weeks) (Mean ± SD) **^**ac**^	38.9 ± 1.4	-	0.001
< 34 (0.9)		-0.10 ± 1.01	
34–36^+6^ (4.4)		0.14 ± 1.07	
≥ 37 (94.7)		-0.01 ± 1.00	

Values represent mean (SD), median (90% range), or number of subjects (%). One-way ANOVA was applied for variance (or the Kruskal–Wallis test) to compare multiple groups, with Bonferroni corrections for post hoc tests. The mothers included in the analyses had a known and reliable date of the first day of the last menstrual period, a regular menstrual cycle of 28 ± 4 days, and a visit between 7 + 0 and 13 + 6 weeks of gestation. Mothers excluded from the analyses had an unknown or unreliable date on the first day of their last menstrual period or an irregular menstrual cycle. Maternal age, pre-pregnancy, and gestational age were divided into three groups. Therefore, we used the same characters to indicate the results of the post-hoc tests. ^a^Group 1 vs. Group 2; ^b^Group 1 vs. Group 3; ^c^Group 2 vs. Group 3

### Risk factors of the CRL in the first trimester

CRL varied with maternal age, pre-pregnancy BMI, parity, folic acid supplement use, maternal vitamin D concentration, foetal sex, and gestational age at delivery (Table 1). In the univariate analyses, higher maternal age, multipara, folic acid supplement use, and higher vitamin D concentration were positively associated with CRL (Table 2). Meanwhile, a higher pre-pregnancy BMI was associated with a shorter CRL. In the multivariate analyses, the associations of maternal age (0.25 mm, 95% CI = [0.22–0.28], *P* < 0.001; 0.04 SDS, 95% CI = [0.03–0.04], *P* < 0.001) per year in age, pre-pregnancy BMI (-0.17 mm, 95% CI = [-0.21 to -0.13], *P* < 0.001; -0.02 SDS, 95% CI = [-0.03 to -0.02], *P* < 0.001) per kg/m^2^ increase in BMI, multipara (0.30 mm, 95% CI = [0.08–0.52], *P* = 0.007; 0.04 SDS, 95% CI = [0.00–0.07], *P* = 0.031), and folic acid supplement use (0.78 mm, 95% CI = [0.49–1.08], *P* < 0.001; 0.05 SDS, 95% CI = [0.01–0.10], *P* < 0.019) with CRL remained significant (Table 2). However, vitamin D concentration was no longer significant.

**Table 2:** Maternal Risk Factors of First-Trimester Variation in Fetal Crown-rump Length Using Univariate and Multivariate Analysis (*N* = 15,524)

	**Univariate analysis**				**Multivariable analysis**			
**Risk Factor**	**Effect Size for Fetal Crown to Rump Length (95% CI), mm**	***P* -Value**	**Effect size of Standard Deviation Score (95% CI)**	***P* -Value**	**Effect Size for Fetal Crown to Rump Length (95% CI), mm**	***P* -Value**	**Effect size of Standard Deviation Score (95% CI)**	***P* -Value**
Maternal Age	0.24 (0.21 to 0.27)	< .001	0.04 (0.03 to 0.04)	< .001	0.25 (0.22 to 0.28)	< .001	0.04 ( 0.03 to 0.04)	< .001
Pre-pregnancy BMI (kg/m^2^)	-0.12 (-0.15 to -0.08)	< .001	-0.02 (-0.02 to -0.01)	< .001	-0.17 (-0.21 to -0.13)	< .001	-0.02 (-0.03 to -0.02)	< .001
Multipara (%)	0.71 (0.50 to 0.93)	< .001	0.10 (0.06 to 0.13)	< .001	0.30 (0.08 to 0.52)	0.007	0.04 (0.00 to 0.07)	0.031
Foliate take	0.77 (0.435 to 1.03)	< .001	0.05 (0.00 to 0.09)	0.044	0.78 (0.49 to 1.08)	< .001	0.05 (0.01 to 0.10)	0.019
Vitamin D	0.01 (0.00 to 0.01)	0.019	0.001 (0.000 to 0.002)	0.020	0.003 ( -0.004 to 0.009)	0.404	0.00 (-0.001 to 0.001)	0.742
Family history of diabetes	-0.30 (-0.68 to 0.07)	0.114	-0.04 (-0.10 to 0.02)	0.163	-	-	-	-

We used foetal sex- and gestational age–adjusted linear regression models to assess the associations of each determinant with first trimester CRL separately. We then presented our results as changes per standard deviation score or actual effect size. Subsequently, all the factors associated with CRL were included in the multivariate generalised linear regression model

### Correlation between CRL and neonatal outcomes

For anatomical parameters, the average birth length was 49.9 ± 1.3 (cm) and the average birth weight was 3342 ± 438 (g) (Table 3). Of the newborns, 3.6% were diagnosed with SGA, 13.8% with LGA, 5.3% were delivered before 37 weeks, and 11.5% were admitted to the NICU. In the univariate analyses, a 1-SDS increase in CRL increased the birth height by 0.08 ± 0.01 (cm) (*P* < 0.001) and the birth weight by 32.78 ± 2.51 (g) (*P* < 0.001). As expected, each SDS increase in CRL was associated with a 25.2% decrease in the odds of SGA (RR = 0.748, 95% CI = 0.687–0.814, *P* < 0.001) and a 19.6% increase in LGA (RR = 1.196, 95% CI = 1.143–1.251, *P* < 0.001). Furthermore, newborns with higher CRL in early pregnancy were more likely to have preterm birth, as a 1-SDS increase in CRL resulted in 9.9% higher odds of preterm birth (RR = 1.099, 95% CI = 1.025–1.179, *P* = 0.008). We also found that CRL was negatively associated with NICU admission rate (RR = 0.933, 95% CI = 0.888–0.979, *P* = 0.005).

**Table 3:** Associations of First-Trimester CRL With Birth Outcomes (*n* = 15 524)

Neonatal Outcomes Total (*N* = 15,524)						
			**Univariable analysis**		**Multivariable analysis**	
	**Mean ± SD or N (%)**	Mean difference (SD)^*^	Effect size per SD or Adjusted RR* (95% CI)	***P* Value**	Effect size per SD or Adjusted RR* (95% CI)	***P* Value**
**Birth length (cm)**	49.9 ± 1.3	-	0.08 ± 0.01	< .001	0.15 ± 0.01	< .001
**Birth weight (g)**	3342 ± 438	-	32.78 ± 2.51	< .001	54.9 ± 2.9	< .001
**SGA**	562 (3.6)	-0.300 (0.043)	0.748 (0.687–0.814)	< .001	0.733 (0.673–0.800)	< .001
**LGA**	2146 (13.8)	0.209 (0.023)	1.196 (1.143–1.251)	< .001	1.241 (1.184–1.301)	0.012
**Preterm birth**^a^	819 (5.3)	0.101 (.036)	1.099 (1.025–1.179)	0.008	1.082 (1.008–1.162)	0.029
**Admission to NICU**	1778 (11.5)	-0.791 (.252)	0.933 (0.888–0.979)	0.005	0.928 (0.883–0.976)	0.003
**Admission to NICU**^**b**^	-	-	-	-	0.934 (0.889–0.982)	0.008

Values represent means (SD) or number of subjects (%). The model was adjusted for maternal age (continuous variable), pre-pregnancy BMI (continuous variable), foliate intake, parity, delivery mode, foetal sex, and gestational age (continuous). ^a^The model of preterm birth was not adjusted for gestational age. ^b^The model of admission to NICU was adjusted for the factors above and SGA

In the multivariate analysis, the associations of birth length [0.15 ± 0.01 (cm) per SDS, *P*_adjusted_ < 0.001], birth weight [54.9 ± 2.9 (g) per SDS, *P*_adjusted_ < 0.001], SGA (RR = 0.733, 95% CI = 0.673–0.800, *P*_adjusted_ < 0.001), and LGA (RR = 1.241, 95% CI = 1.184–1.301, *P*_adjusted_ = 0.012) with CRL were more obvious. Preterm birth (RR = 1.082, 95% CI = 1.008–1.162, *P*_adjusted_ = 0.029) remained significant. A similar result was observed for NICU admission (Model 1: RR = 0.928, 95% CI = 0.883–0.976, *P*_adjusted_ = 0.003). Since SGA newborns were apt to be transferred to the NICU, we included SGA as a covariate in Model 2. The results showed that an increase in SDS was associated with a 6.6% decrease in the NICU admission rate (Model 2: RR = 0.934, 95% CI = 0.889–0.982, *P*_adjusted_ = 0.008). The incidence rates of the other neonatal outcomes are shown in Table S2.

### Associations of first trimester CRL with neonatal outcomes stratified by pre-pregnancy BMI

Pre-pregnancy BMI might be associated with intrauterine malnutrition and several pregnancy complications, resulting in discrepant outcomes. In our study, pre-pregnancy BMI was a risk factor for a lower CRL (-0.17 mm, -0.02 SDS) per kg/m^2^ (Table 2). Therefore, we performed a subgroup analysis and stratified the population based on the pre-pregnancy BMI (Table 4).

**Table 4:** Adjusted risk ratios for neonatal outcomes in CRL levels stratified by maternal BMI (*n* = 15 524)

BMI (kg/m^2^)	Statistic	Birth length	Birth weight	SGA	LGA	Preterm birth^a^	Admission to NICU	
							**Model 1**	**Model 2**^**b**^
** < 18.5 (*n* = 2057)**	**Adjusted RR/ Effect size per SDS**	0.17 ± 0.02	64.74 ± 7.62	0.689 (0.576–0.824)	1.281 (1.057–1.553)	1.477 (1.225–1.780)	0.991 (0.854–1.150)	1.019 (0.877–1.185)
	***P* value**	< .001	< .001	< .001	0.012	< .001	0.902	0.805
**18.5–23.9 (*n* = 11,742)**	**Adjusted RR/ Effect size per SDS**	0.14 ± 0.01	54.2 ± 3.28	0.761 (0.685–0.844)	1.260 (1.193–1.331)	1.053 (0.966–1.147)	0.914 (0.862–0.968)	0.918 (0.866–0.973)
	***P* value**	< .001	< .001	< .001	< .001	0.243	0.002	0.004
** ≥ 24 (*n* = 1725)**	**Adjusted RR/ Effect size per SDS**	0.13 ± 0.03	43.85 ± 8.72	0.679 (0.486–0.949)	1.149 (1.035–1.276)	0.934 (0.782–1.115)	0.940 (0.825–1.072)	0.943 (0.790–1.127)
	***P* value**	< .001	< .001	0.023	0.009	0.448	0.359	0.521

The models were adjusted for maternal age (category), gestational age (weeks), fetus sex, parity (category), mode of delivery

^a^Not included gestational age

^b^Model 2 was adjusted for compound factors in Model 1 and small for gestational age (SGA)

**Fig. 2:**
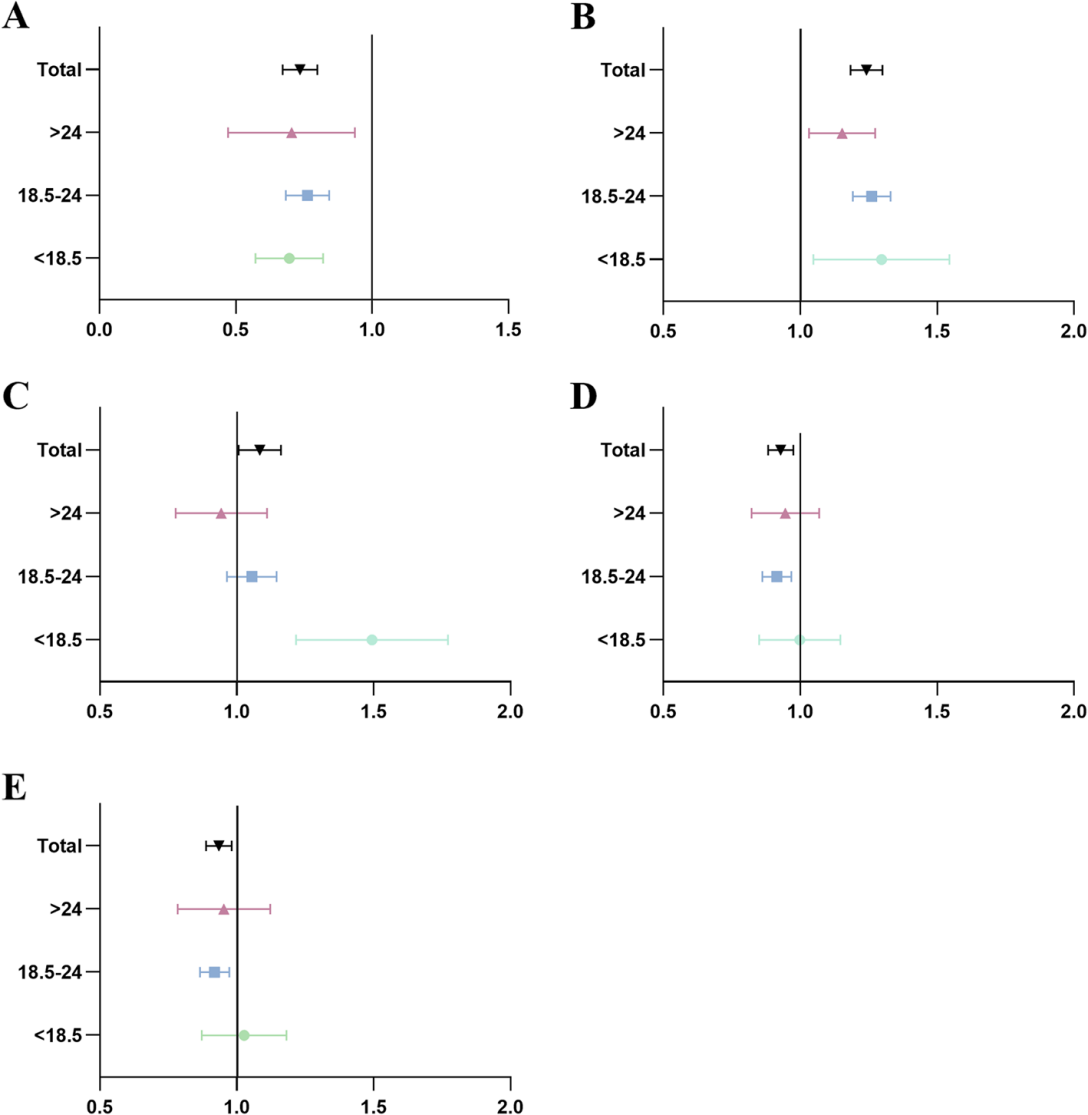
Forest plots of correlation between CRL and neonatal outcomes. **A** SGA. **B** LGA. **C** Preterm birth. **D** NICU (Model 1). **E** NICU (Model 2)

Women with normal BMI (18.5–24 kg/m^2^) had the same trend as previous results for SGA (RR = 0.761, 95% CI = 0.685–0.844, *P*_adjusted_ < 0.001), LGA (RR = 1.260, 95% CI = 1.193–1.331, *P*_adjusted_ < 0.001), and admission to the NICU (Model 1: RR = 0.914, 95% CI = 0.862–0.968, *P*_adjusted_ = 0.002; Model 2: RR = 0.918, 95% CI = 0.866–0.973, *P*_adjusted_ = 0.004). However, an association between preterm birth and CRL was not observed in women with normal pre-pregnancy BMI. In the lean group (< 18.5 kg/m^2^), CRL was strongly related to the incidence rate of SGA, with 31.1% decrease per SDS (RR = 0.689, 95% CI = 0.576–0.824, *P*_adjusted_ < 0.001), and LGA with 28.1% increase (RR = 1.281, 95% CI = 1.057–1.553, *P*_adjusted_ = 0.012). Moreover, 1-SDS increase was associated with a 47.7% increase in the odds of preterm birth (RR = 1.477, 95% CI = 1.225–1.780, *P*_adjusted_ < 0.001). In Figure S2, we used restricted cubic splines to visualise the relationship between the predicted pre-pregnancy BMI and the risk ratio of preterm birth in lean women. The risk ratio was relatively flat until approximately 0 SDS of predicted pre-pregnancy BMI and then started to increase significantly afterwards (*P* for non-linearity = 0.067). Interestingly, for the obesity group (BMI > 24 kg/m^2^), correlations of CRL with neonatal outcomes were only observed in anatomical factors such as birth weight, birth length, SGA, and LGA, where the effect size or RR was relatively smaller than the other two groups. The risk ratios of all outcomes are shown in Fig. 2.

## Discussion

In this study, we investigated the risk factors contributing to CRL, as well as the correlation between CRL in the first trimester and neonatal outcomes. First, maternal age and multipara and folic acid supplement use were positively associated with CRL, whereas pre-pregnancy BMI was negatively related with CRL. Second, CRL was positively associated with birth length, birth weight, incidence rate of LGA, and preterm birth, while it was negatively related to the incidence rate of SGA and NICU admission. Furthermore, when stratified by pre-pregnancy BMI, the correlation between CRL and neonatal outcomes was distinct in the different groups.

Previous studies have indicated that male sex [19], maternal age, and black race [20] are associated with longer CRL in the first trimester. In addition, periconception, alcohol use, smoking [21], and homocysteine [22] are negatively associated with CRL. In our study, we used linear regression adjusted for GA and foetal sex to explore other potential risk factors for CRL. Consistent with other studies, we found that maternal age and use of folic acid supplements were associated with increased foetal growth in early pregnancy. Folic acid participates in several cellular processes, such as cell division, apoptosis [23, 24], and DNA methylation [25], which are involved in foetal and placental development and postnatal growth. In addition, we found that multipara and pre-pregnancy BMI correlated with CRL, which was not observed in other studies [21, 26].

CRL, which serves as an early predictor of foetal growth, is related to neonatal outcomes. Several studies have explored the correlation between first trimester CRL and foetal growth. Leung et al. found that the CRL of the foetus in the first trimester was related to birth weight but was not an independent predictor of SGA in a study of 2,896 cases [27]. A case–control study, including 415 women with singleton pregnancies and at least two CRL measurements, found that infants diagnosed with SGA or LGA at delivery did not show abnormal growth patterns of CRL in the first trimester [28]. Subsequently, a prospective cohort study of 38,033 pregnancies found that the risk of delivering SGA infants decreased with increasing CRL in the first trimester [29]. In our study, CRL was negatively correlated with the incidence rate of SGA and positively correlated with LGA. Our results support the use of CRL as an indicator of foetal growth during early pregnancy.

In addition to foetal size, we found that CRL was slightly yet positively related to preterm birth (RR = 1.082, 95% CI = 1.008–1.162). Inconsistent with our findings, a prospective cohort study indicated that first-trimester growth restriction, defined as less than the 20^th^ percentile of CRL, was associated with an increased risk of preterm birth (adjusted OR = 2.12) [26]. Kwak et al. showed that a small foetal CRL (below the 10th percentile) was associated with an increased risk of preterm delivery before 34 gestational weeks (adjusted OR = 6.48; 95% CI = 1.36–30.79, *P* = 0.019) [30]. Nevertheless, a matched case–control study indicated that a short CRL cannot be used to identify women with an increased risk of preterm birth before 32 weeks of gestation [31]. In the sensitivity analysis, first trimester growth restriction (< 20^th^ percentile of CRL) was not associated with preterm birth in our study (RR = 1.060, 95% CI = 0.889–1.264, *P* = 0.518). Given the inconsistency in the association between CRL and preterm birth, we performed a subgroup analysis based on pre-pregnancy BMI. Interestingly, a correlation between CRL and preterm birth was observed (RR = 1.493, 95% CI = 1.238–1.801) only in the lean group, but not in the normal or obese group. Preterm birth is a complicated process that may be affected by social stress and race [32], infection and inflammation [33], and genetics [34]. In certain circumstances, preterm birth may be evolutionarily advantageous for both mothers and infants. Some studies have indicated that earlier delivery of the foetus may minimise the possibility of cephalopelvic disproportion, and an improper foetal size or head position could prevent the combination of the brain and narrow pelvis [35]. Therefore, we deduced that lean women carrying large CRL foetuses (much more easily developed into LGA and high birth weight afterwards) have an increased risk of preterm delivery, which might be due to protective mechanisms in evolution to avoid potential cephalopelvic disproportion and nutritional limits. However, the deduced viewpoint needs further study on correlation between pelvimetry and CRL.

Interestingly, a larger CRL in the first trimester was a protective factor against admission to the NICU. The trend remained significant only in the normal BMI group, which accounted for most of the population. The SGA was conventionally transferred to the NICU; thus, we constructed another model including the SGA. The trend was retained (RR = 0.915, 95% CI = 0.865–0.968). Admission to the NICU is a comprehensive and robust indicator of newborns’ health.

One of the strengths of our study was its large sample size. The SDS based on ‘GAMLSS’ method was also applied to enable adjustment for gestational age GA to avoid the inclusion of non-linear functions of GA in models. However, owing to the retrospective study design, this study had some limitations. We have not differentiated the aetiology and severity of preterm birth to perform a subgroup analysis, which will be our future interest.

In conclusion, several maternal characteristics contribute to first trimester CRL. CRL might be considered a potential early predictor of foetal size at delivery, preterm birth, and admission to the NICU, since differences in growth trajectories may be expressed in early pregnancy.

## Supplementary Information

**Additional file 1: Figure S1.** Centile curves of CRL measured in 7–13^+6^ gestational weeks fitted with BCGo model (P0.4, P2, P10, P25, P50, P75, P90, P98, P99.6). **Figure S2.** Restricted cubic spline plot of risk ration of preterm birth. **Table S1.** Selection of models of crown-rump length. **Table S2.** Neonatal outcomes of the population.

## Data Availability

The datasets used and/or analysed during the current study are available from the corresponding author on reasonable request.

## Abbreviations

CRL: Crown-rump length
SDS: Standard deviation score
BMI: Body mass index
NICU: Neonatal intensive care unit
GDM: Gestational diabetes mellitus
SGA: Small for gestational age
LGA: Large for gestational age
ICP: Intrahepatic cholestasis of pregnancy

## References

[1] Whitworth M, Bricker L, Mullan C (2015). Ultrasound for fetal assessment in early pregnancy. Cochrane Database Syst Rev.

[2] Ekelund CK, Jørgensen FS, Petersen OB, Sundberg K, Tabor A (2008). Impact of a new national screening policy for Down’s syndrome in Denmark: population based cohort study. BMJ.

[3] Kagan KO, Wright D, Valencia C, Maiz N, Nicolaides KH (2008). Screening for trisomies 21, 18 and 13 by maternal age, fetal nuchal translucency, fetal heart rate, free beta-hCG and pregnancy-associated plasma protein-A. Human reproduction (Oxford, England).

[4] Nicolaides KH, Snijders RJ, Gosden CM, Berry C, Campbell S (1992). Ultrasonographically detectable markers of fetal chromosomal abnormalities. Lancet (London, England).

[5] Smith GC (2004). First trimester origins of fetal growth impairment. Semin Perinatol.

[6] Salomon LJ, Cavicchioni O, Bernard JP, Duyme M, Ville Y (2005). Growth discrepancy in twins in the first trimester of pregnancy. Ultrasound Obstet Gynecol.

[7] Burton GJ, Hempstock J, Jauniaux E (2001). Nutrition of the Human Fetus during the First Trimester—A Review. Placenta.

[8] Genbacev O, Miller RK (2000). Post-implantation Differentiation and Proliferation of Cytotrophoblast Cells: In Vitro Models—A Review. Placenta.

[9] Salomon LJ, Alfirevic Z, Da Silva CF, Deter RL, Figueras F, Ghi T, Glanc P, Khalil A, Lee W, Napolitano R (2019). ISUOG Practice Guidelines: ultrasound assessment of fetal biometry and growth. Ultrasound Obstet Gynecol.

[10] Culliney KA, Parry GK, Brown J, Crowther CA (2016). Regimens of fetal surveillance of suspected large-for-gestational-age fetuses for improving health outcomes. Cochrane Database Syst Rev.

[11] Jarvis S, Glinianaia SV, Torrioli MG, Platt MJ, Miceli M, Jouk PS, Johnson A, Hutton J, Hemming K, Hagberg G (2003). Cerebral palsy and intrauterine growth in single births: European collaborative study. Lancet (London, England).

[12] MacLennan AH, Thompson SC, Gecz J (2015). Cerebral palsy: causes, pathways, and the role of genetic variants. Am J Obstet Gynecol.

[13] Blair EM, Nelson KB (2015). Fetal growth restriction and risk of cerebral palsy in singletons born after at least 35 weeks’ gestation. Am J Obstet Gynecol.

[14] Zhao M, Dai H, Deng Y, Zhao L (2016). SGA as a Risk Factor for Cerebral Palsy in Moderate to Late Preterm Infants: a System Review and Meta-analysis. Sci Rep.

[15] Sparano S, Ahrens W, De Henauw S, Marild S, Molnar D, Moreno LA, Suling M, Tornaritis M, Veidebaum T, Siani A (2013). Being macrosomic at birth is an independent predictor of overweight in children: results from the IDEFICS study. Matern Child Health J.

[16] Whincup PH, Kaye SJ, Owen CG, Huxley R, Cook DG, Anazawa S, Barrett-Connor E, Bhargava SK, Birgisdottir BE, Carlsson S (2008). Birth weight and risk of type 2 diabetes: a systematic review. JAMA.

[17] Lindqvist PG, Molin J (2005). Does antenatal identification of small-for-gestational age fetuses significantly improve their outcome?. Ultrasound Obstet Gynecol.

[18] Ni M, Zhang Q, Zhao J, Shen Q, Yao D, Wang T, Liu Z (2021). Relationship between maternal vitamin D status in the first trimester of pregnancy and maternal and neonatal outcomes: a retrospective single center study. BMC Pediatr.

[19] Bukowski R, Smith GC, Malone FD, Ball RH, Nyberg DA, Comstock CH, Hankins GD, Berkowitz RL, Gross SJ, Dugoff L (2007). Human sexual size dimorphism in early pregnancy. Am J Epidemiol.

[20] Bottomley C, Daemen A, Mukri F, Papageorghiou AT, Kirk E, Pexsters A, De Moor B, Timmerman D, Bourne T (2009). Assessing first trimester growth: the influence of ethnic background and maternal age. Hum Reprod (Oxford, England).

[21] van Uitert EM, van der Elst-Otte N, Wilbers JJ, Exalto N, Willemsen SP, Eilers PH, Koning AH, Steegers EA, Steegers-Theunissen RP (2013). Periconception maternal characteristics and embryonic growth trajectories: the Rotterdam Predict study. Hum Reprod (Oxford, England).

[22] Rubini E, Snoek KM, Schoenmakers S, Willemsen SP, Sinclair KD, Rousian M, Steegers-Theunissen RPM (2022). First Trimester Maternal Homocysteine and Embryonic and Fetal Growth: The Rotterdam Periconception Cohort. Nutrients.

[23] Steegers-Theunissen RP, Smith SC, Steegers EA, Guilbert LJ, Baker PN (2000). Folate affects apoptosis in human trophoblastic cells. BJOG.

[24] Williams PJ, Bulmer JN, Innes BA, Broughton Pipkin F (2011). Possible roles for folic acid in the regulation of trophoblast invasion and placental development in normal early human pregnancy. Biol Reprod.

[25] Crider KS, Yang TP, Berry RJ, Bailey LB (2012). Folate and DNA methylation: a review of molecular mechanisms and the evidence for folate’s role. Adv Nutr (Bethesda, Md).

[26] Mook-Kanamori DO, Steegers EA, Eilers PH, Raat H, Hofman A, Jaddoe VW (2010). Risk factors and outcomes associated with first-trimester fetal growth restriction. JAMA.

[27] Leung TY, Sahota DS, Chan LW, Law LW, Fung TY, Leung TN, Lau TK (2008). Prediction of birth weight by fetal crown-rump length and maternal serum levels of pregnancy-associated plasma protein-A in the first trimester. Ultrasound Obstet Gynecol.

[28] Mongelli M, Lu C, Reid S, Stamatopoulos N, Sankaralingam K, Casikar I, Hardy N, Condous G (2016). Is there a correlation between aberrant embryonic crown-rump length growth velocities and subsequent birth weights?. J Obstet Gynaecol.

[29] Bukowski R, Smith GC, Malone FD, Ball RH, Nyberg DA, Comstock CH, Hankins GD, Berkowitz RL, Gross SJ, Dugoff L (2007). Fetal growth in early pregnancy and risk of delivering low birth weight infant: prospective cohort study. BMJ.

[30] Kwak DW, Yang JI, Song KH, Ryu HM, Han YJ, Kim MY, Chung JH (2022). Prediction of Adverse Pregnancy Outcomes Using Crown-Rump Length at 11 to 13 + 6 Weeks of Gestation. J Ultrasound Med.

[31] Kazemier BM, Kleinrouweler CE, Oudijk MA, van der Post JA, Mol BW, Vis JY, Pajkrt E (2012). Is short first-trimester crown-rump length associated with spontaneous preterm birth?. Ultrasound Obstet Gynecol.

[32] Smid MC, Lee JH, Grant JH, Miles G, Stoddard GJ, Chapman DA, Manuck TA (2017). Maternal race and intergenerational preterm birth recurrence. Am J Obstet Gynecol.

[33] Goldenberg RL, Culhane JF, Iams JD, Romero R (2008). Epidemiology and causes of preterm birth. Lancet (London, England).

[34] Zhang G, Jacobsson B, Muglia LJ (2017). Genetic Associations with Spontaneous Preterm Birth. N Engl J Med.

[35] Muglia LJ, Katz M (2010). The enigma of spontaneous preterm birth. N Engl J Med.

