# Supplementary material for: Correlation between crown-rump length in the first trimester of pregnancy and neonatal outcomes
Source: BMC Pediatr. 2022 Jul 1;22:386. doi: 10.1186/s12887-022-03426-8 (PMC9248167; doi:10.1186/s12887-022-03426-8)
Supplement: Supplementary file 1 — Additional file 1: Figure S1. Centile curves of CRL measured in 7–13+6 gestational weeks fitted with BCGo model (P0.4, P2, P10, P25, P50, P75, P90, P98, P99.6). Figure S2. Restricted cubic spline plot of risk ration of preterm birth. Table S1. Selection of models of crown-rump length. Table S2. Neonatal outcomes of the population. [file 12887_2022_3426_MOESM1_ESM.docx]

**Figure S1**. Centile curves of CRL measured in 7–13^+6^ gestational weeks fitted with BCGo model (P0.4, P2, P10, P25, P50, P75, P90, P98, P99.6).


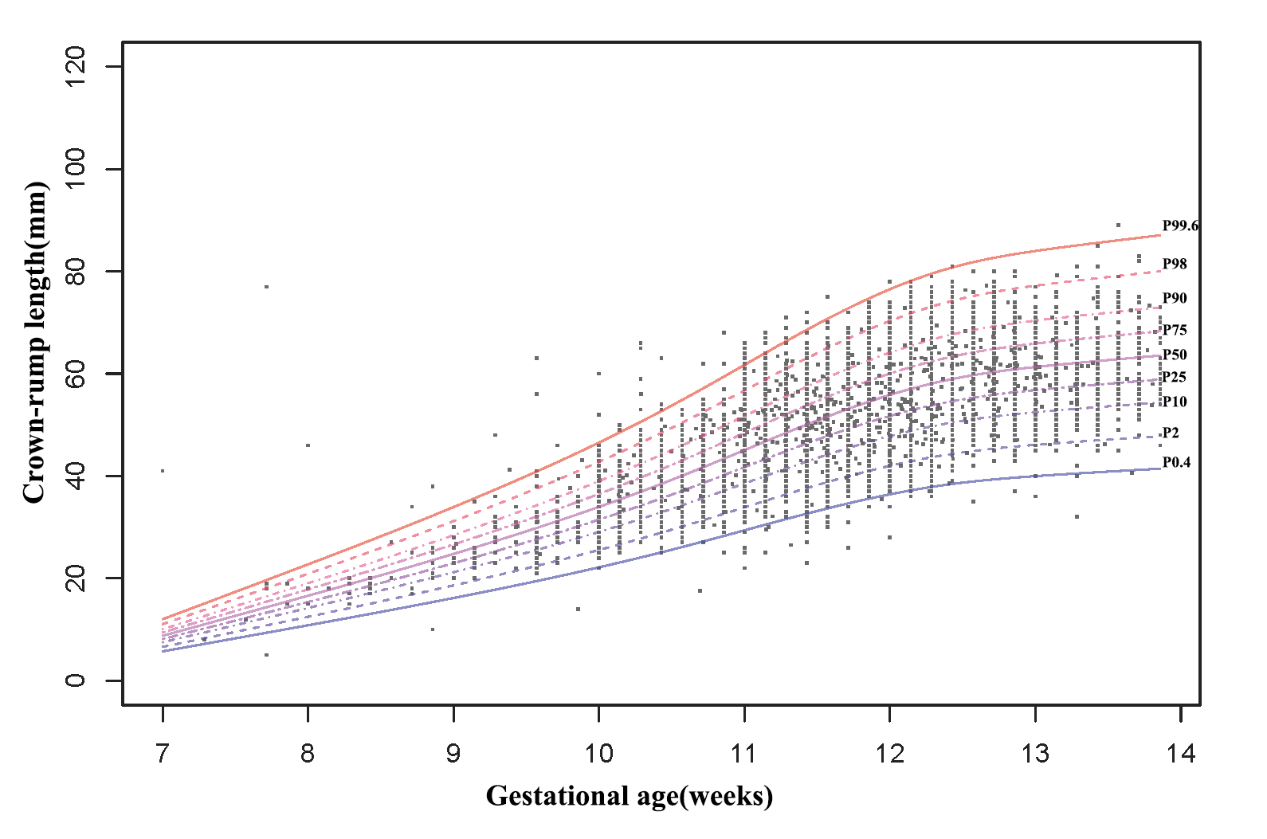


**Figure S2**. Restricted cubic spline plot of risk ration of preterm birth.


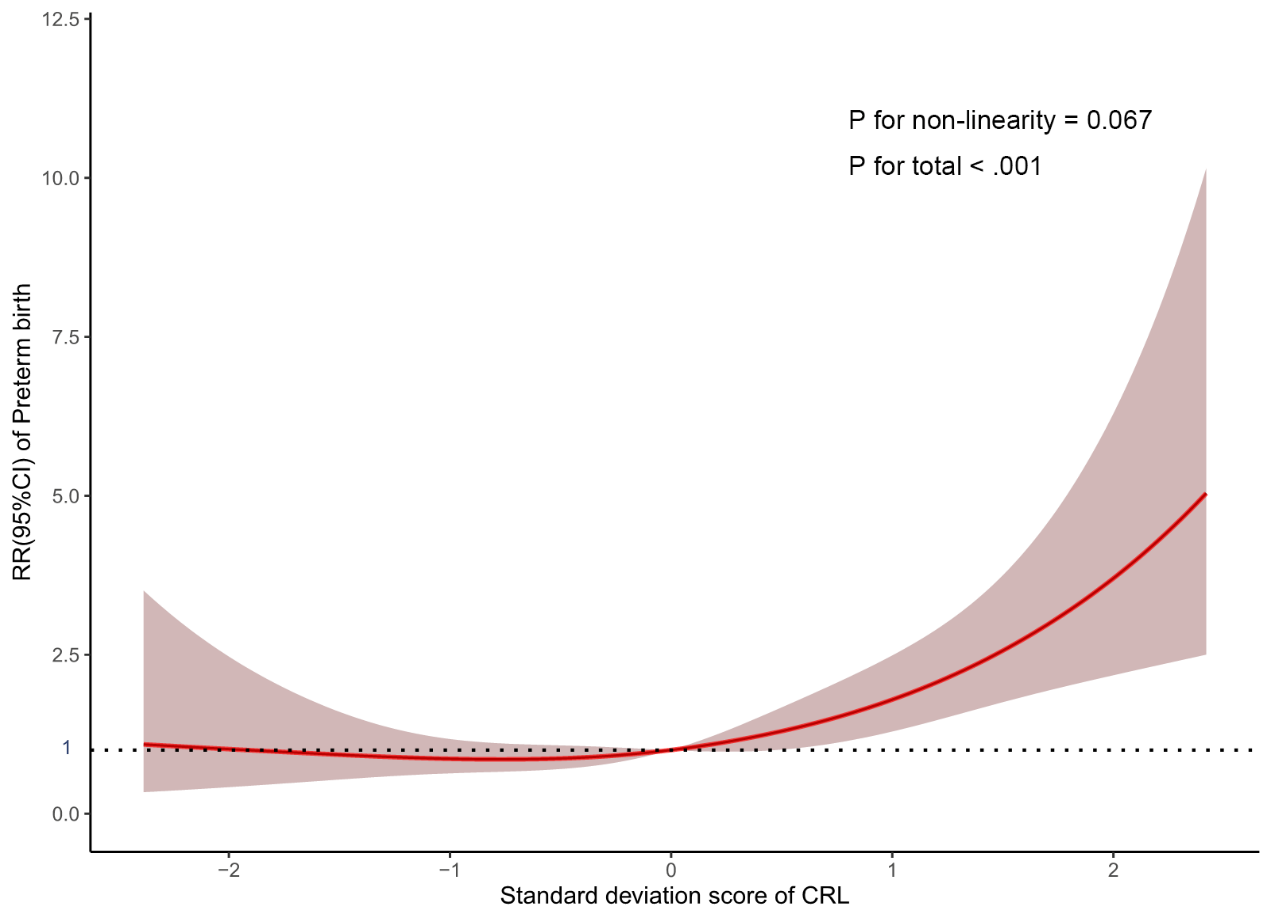


**Table S1 Selection of models of crown-rump length**

| Model | m1 | m2 | m3 | m4 | m5 | m6 | m7 |
| --- | --- | --- | --- | --- | --- | --- | --- |
| Distribution | BCT | BCTo | BCCG | BCCGo | BCPE | BCPEo | Linear regression |
| Cycle | 16 | 10 | 13 | 13 | 15 | 9 | - |
| df | 8 | 8 | 7 | 7 | 8 | 8 | 1 |
| Global deviance | 100870.5 | 100800.1 | 101689.6 | 101670.8 | 101180.9 | 101118.1 | - |
| AIC | 100886.5 | 100816.1 | 101703.6 | 101684.8 | 101196.9 | 101134.1 | 69448 |
| SBC | 100947.7 | 100877.3 | 101757.2 | 101738.3 | 101258.1 | 101195.4 | - |
| Mean/median | 0 | 0 | 0.03^*^ | 0.01^*^ | 0.03^*^ | 0.00^*^ | 0 |
| SD | 1 | 1 | - | - | - | - | 6.55 |
| Skewness | 0.01 | 0.01 | - | - | - | - | 0.24 |
| Kurtosis | 3.18 | 3.14 | - | - | - | - | 5.33 |

* The data was presented as median.

**Table S2. The neonatal outcomes of the population.**

| **Neonatal outcomes (n = 15 524)** | **N (%)** |
| --- | --- |
| **Hyperbilirubinemia** | 909(5.9) |
| **Wet lung** | 249(1.6) |
| **Asphyxia** | 122(.8) |
| **Hypoglycemia** | 67(.4) |
| **Necrotizing Enterocolitis** | 65(.4) |
| **Respiratory distress syndrome** | 22(.1) |
| **Pneumonia** | 43(.3) |
| **Convulsion** | 8 |
| **Sepsis** | 25(.2) |
| **Retinopathy** | 13(.1) |
| **Still birth** | 22(.1) |
| **Hyperbilirubinemia** | 909(5.9) |
